# Effects of kinesiotherapy, ultrasound and electrotherapy in management of bilateral knee osteoarthritis: prospective clinical trial

**DOI:** 10.1186/1471-2474-13-182

**Published:** 2012-09-22

**Authors:** Naryana Cristina Mascarin, Rodrigo Luiz Vancini, MarÃ­lia dos Santos Andrade, Eduardo de Paiva Magalhães, Claudio Andre Barbosa de Lira, Ibsen Bellini Coimbra

**Affiliations:** 1 Departamento de Fisiologia, Universidade Federal de São Paulo (UNIFESP), Rua Botucatu, 862, 5° andar – Ed. Ciências Biomédicas, Vila Clementino, CEP: 04023-900, São Paulo, (SP), Brazil; 2 Departamento de Clínica Médica, Faculdade de Ciências Médicas Universidade Estadual de Campinas (UNICAMP), SP, Brazil, Avenida Alexander Fleming,181, 2°. piso, sala 07, Barão Geraldo, CEP: 13083-881, Campinas, Caixa-Postal: 6111, (SP), Brazil; 3 Setor de Fisiologia Humana e do Exercício, Universidade Federal de Goiás (UFG), Unidade Jatobá, Câmpus Jataí, Rod. BR 364, km 192, Parque Industrial, no. 3800, CEP: 75801-615, Jataí, GO, Brazil

**Keywords:** Kinesiotherapy, Ultrasound, Electrotherapy, Knee osteoarthritis

## Abstract

**Background:**

Although recent advances in knee osteoarthritis (OA) treatment and evaluation were achieved, to the best of our knowledge, few studies have evaluated the longitudinal effect of therapeutic modalities on the functional exercise capacity of patients with knee OA. The purpose was to investigate the effects of kinesiotherapy and electrotherapy on functional exercise capacity, evaluated using the six-minute walk test (6-MWT) in patients with bilateral knee OA. Secondary measurements included range of motion (ROM), severity of knee pain (VAS), and a measure of perceived health and physical function, evaluated using the Western Ontario and McMaster Universities (WOMAC) Osteoarthritis Index.

**Methods:**

A total of 40 women with bilateral knee OA were assigned to three groups: kinesiotherapy (KIN, *n* = 16), transcutaneous electrical nerve stimulation (TENS, *n* = 12), or ultrasound (US, *n* = 10). The groups underwent 12 weeks of intervention twice per week. The participants were subjected to the 6-MWT, ROM, VAS and WOMAC index. These tests were performed before and after the intervention. The study was focused on outpatients and was carried out at Universidade Estadual de Campinas, Brazil.

**Results:**

At follow-up, the KIN and US groups had significantly higher 6-MWT distances (19.8 ± 21.7 and 14.1 ± 22.5%, respectively) compared with their respective pre-intervention values. All treatments were effective for reducing pain and improving the WOMAC index.

**Conclusions:**

We demonstrated that the 6-MWT is a tool that can be used to evaluate improvements in the functional exercise capacity of patients submitted to a clinical intervention.

## Background

Osteoarthritis (OA) is a chronic and degenerative joint disease and is considered one of the most common musculoskeletal disorders [1,2]. Approximately 85% of the population near 65 years of age present radiographic evidences of OA [3]. The knees, hands, hips, spine, and feet include the joints most often affected by OA [4-6]. The main clinical symptoms related by patients with knee OA include pain, articular stiffness, crepitation, articular edema, joint deformities, articular instability, decrease in range of motion (ROM), physical activity limitations and muscle weakness [6,7]. For these reasons, several pharmacologic [8,9] and non-pharmacologic strategies [10-13] have been studied for relief of knee pain.

Physiotherapy is one of the professions that provide effective non-pharmacological interventions for people with knee OA [14] and procedures prescribed by physiotherapists are considered important and play a fundamental role in patient treatment. In this context, kinesiotherapy (KIN), which comprises different types of therapeutic exercises, such as stretching, strengthening (isotonic, isokinetic, and isometric) and aerobic exercise, [15] and electrotherapy are frequently used for the treatment of different musculoskeletal disorders [16-18]. The most common types of electrotherapy are ultrasound (US), a modality of treatment that uses sound waves to generate heat within a body part, and transcutaneous electrical nerve stimulation (TENS), a method of pain relief in which a special device transmits low-voltage electrical impulses through electrodes on the skin to an area of the body that is in pain [4].

Despite recent advances in OA treatment, few studies have evaluated the longitudinal effect of therapeutic modalities on the functional capacity of patients with knee OA, especially that functional capacity related to exercise performance. Lin et al. [19] described the results of a battery of physical function tests used to assess the physical function of older patients with clinical knee and/or hip OA. These tests included: walk a distance of 8 feet, ascend/descend 4 stairs, and stand and sit on a chair 5 times. The authors stated that these physical function tests are safe, practical, and may be useful in the evaluation of therapeutic interventions. French et al. [20] compared the responsiveness of three physical performance measures of function following physiotherapy for OA of the knee and found that the 6-min walk test (6-MWT) was more responsive in the assessment of physical performance than the timed-up-and-go test and the timed-stand test. In this context, the 6-MWT is a simple, safe and low-cost field test often used to evaluate chronic heart failure patients, chronic obstructive pulmonary disease patients, and the elderly to regularly assess functional exercise capacity and the effects of a rehabilitation/exercise program [21,22].

Therefore, the primary purpose of this study was to investigate the effects of 12 weeks of kinesiotherapy and electrotherapy on functional exercise capacity as evaluated by the 6-MWT. Secondary measurements included range of motion, severity of knee pain, and the measure of perceived health and physical function evaluated by the Western Ontario and McMaster Universities (WOMAC) Osteoarthritis Index [23,24].

## Methods

### Participants

Patients with knee OA were recruited from a Rheumatology Clinic. These patients were initially contacted, evaluated, and informed about the objective and experimental procedures of the study. Of the 48 patients initially recruited, 40 patients completed all experimental procedures. Exclusion criteria included any rheumatic disease (with the exception of bilateral knee OA), unilateral knee OA, neurological disorders, cognitive limitations or history of cardiovascular, pulmonary or endocrinology disease. Inclusion criteria included female gender, a minimum of 45 years old, free from any other lower limb disease (except bilateral knee OA), able to perform physical exercise, not currently receiving physical therapy treatments for the knee OA condition, medication compliance (all patients were taking glucocorticoids at the time of study), and diagnosis of bilateral knee OA according to the American College of Rheumatology criteria [25]. The participants were randomly divided into three groups: kinesiotherapy (KIN, n = 16), transcutaneous electric nerve stimulation (TENS, n = 12), and ultrasound (US, n = 12). These physiotherapy interventions were performed twice per week for 12 weeks. The patient characteristics are presented in Table 1. 

**Table 1:** Characteristics of the subjects

	**Experimental groups**		
	**KIN (n = 16)**	**TENS (n = 12)**	**US (n = 12)**
Age (yr)	59.6 ± 7.2	64.8 ± 7.0	62.8 ± 7.6
	(48 – 70)	(50 – 74)	(51 – 77)
Height (cm)	154.6 ± 6.1	153.4 ± 6.8	153.8 ± 6.0
	(146.0 – 166.0)	(143.0 – 165.0)	(141.0 – 163.0)
Body mass (kg)	71.1 ± 10.8	73.9 ± 13.7	71.3 ± 10.0
	(48.0 – 92.0)	(58.0 – 112.0)	(50.9 – 85.0)
Years diagnosed with osteoarthritis	5.6 ± 5.6	5.2 ± 6.8	4.8 ± 3.4
	(0.08 – 15)	(1 – 25)	(1 – 10)

Data expressed as mean ± S.D. (min – max).

### Experimental design

The study was organized in four successive phases: a basal medical and physical examination, the pre-intervention evaluations, the treatment period, and the post-intervention evaluations. The basal medical examination was performed three days before the beginning of the treatment period. The participants underwent a detailed medical examination (performed by a rheumatologist) and OA diagnostic evaluation (based on symptoms and conventional standing antero-posterior knee radiographs). In the two days before and after the treatment intervention, all of the participants performed follow-up evaluations in this order: perceived health and physical function by Western Ontario and MacMaster Universities Osteoarthritis Index (WOMAC) Questionnaire, pain by using a visual analogical scale (VAS), ROM by using a goniometer, and functional exercise capacity evaluated by the 6-MWT. Finally, the participants were submitted to 12 weeks of treatment intervention, twice per week, on non-consecutive days.

The participants were instructed to arrive at the laboratory in a rested and fully hydrated state, having not consumed caffeine in the previous 4 h, and to avoid strenuous exercise in the 48 h preceding a session. To minimize the effects of diurnal biological variation, all the tests were performed at the same time of day.

All experimental procedures were approved by the University Human Research Ethics Committee and conformed to the principles outlined in the Declaration of Helsinki. All participants signed informed consent forms prior to participating in the study.

### Modes of physiotherapy treatment

The participants in each group participated in their respective treatment intervention for 12 weeks (24 sessions). All sessions were supervised by an experienced physical therapist. Missed sessions were compensated during the subsequent weeks so that the total number of sessions was completed.

The KIN protocol consisted of supervised stretching and isometric exercises for the entire lower limb. The stretching exercises were performed actively, using the static method. The participants were instructed to perform three bouts of 30 seconds each in each lower limb to the following muscles and in this order: calf, quadriceps, and hamstring muscles. The stretches were alternated for each limb. The static stretching exercises were performed until the maximal range of motion or pain threshold was reached. The isometric exercises consisted of three exercises using a conventional plastic ball (diameter of 20 cm) and one exercise using an elastic band (Rubber Band, Orange Color, Carci, Brazil) with extra strong resistance measuring 1.50 x 0.14 m. The participants were instructed to perform a total of 30 repetitions. Each repetition lasted 6 seconds with an interval of approximately 3 seconds. In the first exercise using the ball, the patients were placed in a supine position with knees flexed. The ball was positioned between the patient’s knees, and the patient was instructed to press the knees against the ball to perform a maximal contraction. This exercise aimed to strengthen the adductor muscles. In the second exercise using the ball, the patients were placed in a supine position with one knee flexed and the other knee in full extension. With the ball placed under the ankle of the limb that was extended, the participants performed a maximal contraction against the ball. The patient alternated performing the exercise for each lower limb, and this exercise aimed to strengthen the quadriceps muscles. In the third exercise using the ball, the patient was positioned prone with both knees extended. The ball was placed under one ankle, and the patient was instructed to perform a maximal contraction against the ball. The patient alternated performing the exercise for each lower limb, and this exercise aimed to strengthen the hamstring muscles. Finally, in the fourth exercise, the patients were placed in a supine position with knees flexed. The knees were tied with an elastic band, and the patients were requested to perform a maximal abduction movement of the lower limbs. This exercise aimed to strengthen the abductor muscles. Each session lasted approximately 20 minutes.

The TENS was delivered by a transcutaneous electrical stimulator (Neurodyn II, Ibramed, Brazil) with two channels and four square, self-adhesive percutaneous electrodes measuring 5 x 5 cm. The TENS was applied using a frequency of 100 Hz, pulse width of 50 μs, intensity (mA) set at the individual subject's sensorial threshold, modulation up to 50% of variation frequency, quadratic biphasic symmetrical pulse and a length of application of 20 minutes. In the TENS protocol, the participants were stimulated in dorsal decubitus, adequately positioned with a roll under their knees. The percutaneous electrodes for the electrical stimulation were placed on the anterior medial and lateral portions of the knee. This group also performed the same stretching and isometric exercises for the lower limbs described for the KIN group. Each session lasted approximately 40 minutes.

The US protocol consisted of continuous ultrasonic waves of 1 MHz frequency and 0.8 W/cm [2] power, applied with a 5-cm diameter applicator (Sonic, 1–3 MHz, HTM, Brazil). The patients were placed in a supine position, and an acoustic gel that did not contain any pharmacologically active substance was applied. Ultrasound was then applied to the medial and lateral parts of the knee in circular movements with the probe at right angles to ensure maximum absorption of the energy. Each session lasted 3–4 minutes, depending on the knee size due to edema. During the evaluation, we observed that some subjects exhibited evidence of edema. This group also performed the same stretching and isometric exercises for the lower limbs described for the KIN group. Each session lasted approximately 25 minutes.

### Assessments

The participants were assessed at baseline and at the end of the treatment by an investigator who was blind to the randomization. The following assessments were performed: severity of pain, ROM for extension and flexion of the knee, the 6-MWT, and the perceived health and physical function.

### Severity of knee pain

Knee pain was assessed using a VAS. The VAS consists of a 10‒cm line, with the left extreme indicating “no pain” or zero and the right extreme indicating “unbearable pain” or 10. The participants were asked to use the scale to indicate their current level of pain. Higher values suggest more intense pain. The values (in centimeters) were recorded for the statistical analysis.

### Range of motion (ROM)

Knee flexion and extension ROM in degrees were measured bilaterally in a supine position according to Norkin and White [26]. To this end, the lateral femoral condyle was used as a landmark for the measurement of knee flexion and extension. The central pivot of a universal goniometer (CARCI, São Paulo, Brazil) was placed over the midpoint of the lateral joint margin, with the stationary arm of the universal goniometer aligned with the great trochanter. The moving arm of the goniometer was then aligned with the lateral malleolus with the neutral position taken as zero. For the knee flexion measurement, initially the hip was at zero degrees of extension, abduction, and adduction, but as the patients maximally flexed the knee, the hip also flexed. Thus, the examiner supported the lower limb and stabilized the femur to prevent rotation, abduction, and adduction of the hip. For the knee extension, the measurement was made with the lower limb extended. The previous precautions to prevent compensations (i.e., adduction, abduction, and rotation) were taken. The measurements were performed by two experienced physical therapists. Each knee and position (flexion or extension) was measured twice, and the higher angle was recorded for the statistical analysis.

### The six-minute walking test (6-MWT)

The 6-MWT was performed to evaluate functional exercise capacity in a 100 m-long indoor hallway free of obstacles. The length of the corridor was marked every 1 m. The participants were instructed to walk at a self-selected regular pace to cover as much distance as they could during the allotted time. If necessary, slowing down and stopping to rest were allowed. At the end of each minute, the participants were given feedback on the elapsed time and standardized encouragement in the form of statements such as “you are doing well, keep it up” and “do your best.” These technical aspects are in line with the American Thoracic Society recommendations for the 6-MWT [27]. The distance covered (in meters) was used for the statistical analysis. The test-retest reliability of the 6-MWT has been ascertained in patients with knee OA [28]. During the test, all the participants walked independently without using walking aids.

### WOMAC Osteoarthritis Index

A disease-specific index of disability, the WOMAC Osteoarthritis Index, was used as a subjective measure of perceived health and physical function. The WOMAC Osteoarthritis Index is a three-part questionnaire that can be completed by the subject in approximately 10 minutes, consists of 24 questions and probes clinically important symptoms in the areas of pain (5 questions), stiffness (2 questions), and physical function (17 questions) for patients with OA of the hip and/or knee [23,24]. In the present study, we used a Likert scale version of the WOMAC that allows patients to make their responses on a five-point scale (0 = none, 1 = mild, 2 = moderate, 3 = severe, 4 = extreme). The higher the score achieved, the lower the level of perceived health and physical function. Scores were generated for the three dimensions of pain, stiffness and physical function by summing the coded responses. The patient should answer the questions to best describe their symptoms and difficulties from the past 72 hours [29]. Psychometric studies have shown moderate to high validity and reliability for the WOMAC questionnaire [30].

### Statistical analysis

STATISTICA v 7.0 for Windows was used for the statistical analyses. All the variables presented normal distributions according to Kolmogorov-Smirnov tests. Two-way repeated-measure analyses of variance (ANOVA) were used to assess group (KIN *vs.* TENS *vs.* US) and time (before vs. after) differences in the variables measured. When significant group-by-time interactions were present, Tukey’s post hoc procedures were used to identify the specific differences.

To describe the differences in related treatments, the effect sizes were calculated as the difference between the means divided by the pooled standard deviation. On the basis of Cohen’s criteria (29), an effect size of ≥0.20 and <0.50 was considered small, ≥0.50 and <0.80 medium, and ≥0.80 large.

All the data are presented as the mean ± standard deviation (SD) (min – max). The results were determined to be statistically significant at p < 0.05.

## Results

All the participants completed 24 treatment sessions. Table 2 presents the data with respect to the evaluation of right and left knee joint pain by VAS (cm) of the KIN, TENS and US groups. No significant differences were observed between the groups before the treatment period for the right and left knee (mean for all groups: 7.4 ± 1.9 cm) thus, there is no superiority in comparison between the different types of treatment. However, in the intra-group comparisons (before *vs.* after) a significant decrease was observed in the VAS for pain in all experimental groups and for both knees except for the left knee in the US group. No significant differences were observed between the groups for the right and left knee after the treatment period.

**Table 2:** Visual analog score (in centimeter) for both knees in each group before and after treatment

	**Right Knee**				**Left Knee**			
	**Before**	**After**	***P***	**Effect size**	**Before**	**After**	***P***	**Effect size**
KIN (n = 16)	6.9 ± 1.9 (5.0 – 10.0)	2.3 ± 2.7^a^ (0.0 – 8.0)	0.0001	0.70	7.0 ± 2.1 (4.0 – 10.0)	2.4 ± 2.8^a^ (0.0 – 7.0)	0.0008	0.68
TENS (n = 12)	8.0 ± 1.5 (6.0 – 10.0)	2.6 ± 2.9^a^ (0.0 – 7.5)	0.0001	0.76	5.6 ± 2.7 (0.0 – 10.0)	2.3 ± 2.5^a^ (0.0 – 9.0)	0.004	0.53
US (n = 12)	6.6 ± 3.0 (0.0 – 10.0)	4.5 ± 3.7^a^ (0.0 – 10.0)	0.009	0.41	7.3 ± 2.3 (4.0 – 10.0)	3.8 ± 3.1 (0.0 – 7.0)	0.054	0.54

Data expressed as mean ± S.D. (min – max).

^a^ different from before for the same group.

The data obtained from the evaluation of knee ROM are presented in Table 3. No significant differences were observed between the groups before the treatment period for the right and left knee in flexion and extension thus, there is no superiority in comparison between the different types of treatment. The protocols adopted by the present study did not cause improvements in flexion for either knee. For extension, increases in ROM were found in the KIN and TENS groups for both knees.

**Table 3:** Range of motion (in degrees) for both knees in each group before and after treatment

	**Flexion**						**Extension**					
	**Right**			**Left**			**Right**			**Left**		
	**Before**	**After**	***P***	**Before**	**After**	***P***	**Before**	**After**	***P***	**Before**	**After**	***P***
KIN	76 ± 9	73 ± 12	>0.77	74 ± 11	69 ± 12	>0.98	171 ± 6	177 ± 4^a^	0.0003	172 ± 5	178 ± 3^a^	0.001
(n = 16)	(56 – 90)	(55 – 90)		(50 – 90)	(55 – 87)		(160 – 180)	(168 – 180)		(161–180)	(170 – 180)	
TENS	79 ± 7	76 ± 10	>0.34	81 ± 12	79 ± 7	>0.44	172 ± 6	178 ± 3^a^	0.003	170 ± 8	176 ± 4^a^	0.002
(n = 12)	(68 – 90)	(59 – 90)		(50 – 90)	(65 – 89)		(160 – 180)	(170 – 180)		(150 – 180)	(168 – 180)	
US	81 ± 8	76 ± 7	>0.83	80 ± 8	75 ± 8	>0.39	171 ± 6	175 ± 7	0.21	172 ± 7	173 ± 7	0.47
(n = 12)	(67 – 90)	(66 – 87)		(67 – 90)	(56 – 86)		(165 – 180)	(156 – 180)		(160 – 180)	(160 – 180)	

Data are expressed as mean ± S.D (min – max).

^a^ different from before for the same group.

The WOMAC total scores and the score for each dimension were similar in all three groups at baseline except for the KIN group when compared with the US group (Table 4). Compared with the baseline, significant improvements were observed in each group at the end of the treatment. The improvement in the patients treated with US was significantly less pronounced than that in the patients from the KIN and TENS groups (p < 0.05).

**Table 4:** Scores for the WOMAC Index in each group before and after treatment

	**Pain**			**Rigidity**			**Physical function**			**Total score**			
	**Before**	**After**	***P***	**Before**	**After**	***P***	**Before**	**After**	***P***	**Before**	**After**	***P***	**Effect size**
KIN (n = 16)	8.9 ± 4.4 (1 – 18)	2.0 ± 2.3^a^ (0 – 8)	0.0001	3.0 ± 2.1 (0 – 6)	0.4 ± 0.8^a^ (0 – 2)	0.0001	25.6 ± 13.6^b^ (6 – 48)	4.6 ± 5.9^ab^ (0 – 21)	0.0001	37.5 ± 18.7^b^ (7 – 69)	7.0 ± 8.1^ab^ (0 – 28)	0.0001	0.73
TENS (n = 12)	10.7 ± 3.0 (4 – 15)	3.3 ± 2.9^a^ (0 – 9)	0.0001	4.3 ± 1.9 (2 – 8)	0.8 ± 0.8^a^ (0 – 2)	0.0001	31.8 ± 9.2 (16 – 50)	10.1 ± 8.3^ab^ (0 – 25)	0.0001	46.8 ± 12.2 (22 – 69)	14.2 ± 11.0^ab^ (0 – 35)	0.0001	0.81
US (n = 12)	10.1 ± 3.8 (4 – 16)	6.2 ± 4.2^a^ (2 – 17)	0.01	4.4 ± 2.5 (0 – 8)	2.0 ± 1.9^a^ (0 – 6)	0.004	38.3 ± 9.1 (22 – 51)	20.6 ± 9.8^a^ (5 – 43)	0.0001	53.5 ± 12.2 (36 – 70)	28.8 ± 14.8^a^ (8 – 66)	0.0002	0.67

Data are expressed as mean ± S.D. (min – max).

^a^ different from before for the same group.

^b^ different from US group at same time point.

The 6-MWT was completed by all the subjects without premature cessation and/or breaks. No symptoms or clinical complications occurred during the tests. The 6-MWT performances of the participants are shown in Table 5. No statistically significant differences between the groups were found at baseline. The distances completed and the walking speeds were significantly higher in the KIN (19.8%) and US (14.1%) groups when compared to the pre-treatment values. No statistically significant difference was found in the TENS group (8.9%). No statistically significant differences between the groups were found after the treatment period.

**Table 5:** Distance covered and percent change obtained in the six-minute walk test before and after treatment

	**Before**	**After**	**Δ%**	***P***	**Effect size**
KIN (n = 16)	333 ± 80	387 ± 59^a^	19.8 ± 21.7	0.003	−0.35
	(212 – 500)	(308 – 500)	(−6.1 – 74.5)		
TENS (n = 12)	330 ± 61	355 ± 65	8.9 ± 17.1	0.61	N/A
	(200 – 420)	(275 – 500)	(−22.9 – 37.5)		
US (n = 12)	318 ± 68	358 ± 77^a^	14.1 ± 22.5	0.04	−0.26
	(200 – 400)	(200 – 450)	(−6.3 – 80.0)		

Data are expressed as mean ± S.D. (min – max).

^a^ different from before for the same group. N/A: not applicable.

## Discussion

Knee OA is expected to be the fourth highest cause of disability in women and is responsible for the deterioration of quality of life and functional capacity [31]. A plethora of studies have investigated several aspects related to muscle function, such as strength [32] and aerobic capacity [33] as well as other clinical aspects such as pain [34], stiffness [35], ROM [36] and WOMAC index [37] in patients with OA. Despite these important advances, to our knowledge, few studies have investigated the effects of different types of non-pharmacological treatments on the functional exercise capacity of patients with OA. In this context, the 6-MWT is an excellent tool to evaluate the effect of therapy on the functional exercise capacity. In this study, we found that the KIN and US procedures improved the functional exercise capacity of patients with bilateral knee OA after the intervention period; however, we found no inter-group differences. Moreover, we also evaluated the effect of the treatment period on pain using the VAS and WOMAC index, and we found that the three interventions improved the pain. The difference in this study is that our sample is homogeneous because we recruited only women with bilateral knee OA.

Pain is one of the most common complaints and disabling symptoms in OA populations. In the present study, we evaluated the efficacy of different treatment modes on knee pain, measured using the VAS and the pain dimension of the WOMAC index. We found that pain in both knees decreased in all the experimental groups. This is not the first study to demonstrate the positive effects of non-pharmacologic management on knee pain in OA patients. The Cochrane group [38] systematically reviewed and combined the study results of 17 OA exercise studies (a total of 2562 participants). This group found that land-based exercise had a small-to-moderate beneficial effect on pain for people with symptomatic knee OA. Roddy et al. [39] reviewed 19 randomized clinical trials investigating the effects of land-based exercise for knee or hip OA. They concluded that both strengthening and aerobic exercises performed on land could reduce pain and improve the function and health status in patients with knee and hip OA. However, these authors stated that there was not enough evidence to support or recommend specific types of exercise.

Concerning the TENS, Rutjes et al. [5] conducted a systematic Cochrane review of transcutaneous electrostimulation vs. sham or no specific intervention on pain in individuals with knee OA. This systematic review found little evidence of a significant effect for electrostimulation compared to sham or no intervention on pain in knee OA. The authors attributed these results to the poor quality of the trials and the high degree of heterogeneity across the studies. Our results contradict this systematic review because we found an improvement in the pain index (VAS and pain dimension of the WOMAC index) in all the experimental groups. To evaluate the therapeutic effect of the TENS modalities, NG et al. [40] studied 24 patients and compared electroacupuncture treatment and TENS, using the same parameters for both (low frequency - 2 Hz, continuous mode, pulsation of 200 μs for 20 min of application, and a control group with only educational orientations on OA of the knee) and showed that either electroacupuncture treatment or TENS are effective in pain reduction because a prolonged analgesic effect was maintained in the two groups. Another study was performed with 62 patients between 50 and 75 years of age and presenting knee OA during a four-week period. These patients were divided into four treatment groups: TENS placebo group, TENS group, exercise group and TENS plus exercise group. The results showed no significance between the different types of treatment due to the protocol duration [41]. This treatment was similar to ours in the number of patients and the modalities used, such as the conventional TENS and the isometric exercises. However, the TENS parameters and the application time were different and, unlike our study, did not present significance in the protocols due to the short treatment duration. In our study, the three groups (KIN, TENS and US) showed significant differences after the treatment duration.

In regards to the US, Loyola-Sanchez et al. [42] conducted a meta-analysis of the efficacy of US for decreasing pain and improving physical function in people with knee OA. New evidence was found that shows that US can reduce pain by 21% compared to a control group.

Range of motion was another variable evaluated in the present study. We did not observe any difference due to the three modes of treatment used in this study. Our results agree with Tascioglu et al. [43] who compared the effectiveness of ultrasound (continuous versus pulsed) therapy versus placebo ultrasound in patients with knee OA and also found no differences in ROM. These authors also found improvements in the WOMAC index and functional capacity as evaluated by a 20-m walking test.

Finally, we were particular interested in evaluating the effect of KIN, US and TENS treatment on the 6-MWT performance of woman with bilateral knee OA. Several modalities are available for the objective evaluation of cardiorespiratory fitness. Some provide a very complete assessment of all the systems involved in exercise performance, whereas others provide basic information but are low-tech and easy to perform. The 6-MWT is a simple test that requires a hallway but no equipment or advanced training for the technicians. This test evaluates the global and integrated responses of all the systems involved during exercise, including the pulmonary, cardiovascular and muscular systems [22]. To help predict the total distance walked during the 6-MWT, Enright and Sherrill [44] established a reference equation that incorporates subject characteristics such as age, body mass and height. These subject characteristics were shown to be associated with the distance walked during the 6-MWT. When applying this reference equation to the current data, the results revealed that the KIN, US and TENS groups walked 74%, 79% and 85%, respectively, of the predicted values found by the Enright and Sherrill [44] equation in the pre-evaluation. These modest values demonstrate the low functional exercise capacity, and consequently low health status, of the patients evaluated in the present study. On average, our patients walked 328.8 m before the treatment. These values agree with Wang et al. [45] who compared the efficacy of aquatic exercises and land-based exercises for patients with knee OA. However, these values are lower than those reported by French et al. [20] (405.1 m). The difference most likely results from the poorer physical condition of our volunteers, as represented in the lower highest total score obtained for the WOMAC index compared with that in the study by French et al. [20] Additionally, the sample studied by French et al. [20] contained male participants with unilateral and bilateral knee OA. The difference in this study is that our sample is homogeneous because we recruited only women with bilateral knee OA.

The impact of health status on 6-MWT performance was investigated in 165 elderly people. The covered distance decreased significantly with increasing age and with worsening health status (corrected for age) [46]. Patients with dilated cardiomyopathy were also investigated, and the results demonstrated that the covered distance and peak oxygen uptake (cardiorespiratory fitness index) were closely correlated [47]. In addition, the authors found a correlation between the 6-MWT covered distance and the New York Heart Association functional class. Santana et al. [21] showed that in the healthy elderly, the 6-MWT can be used to evaluate improvements in functional exercise capacity after exercise training. However, the 6-MWT is not appropriate to evaluate improvements in the cardiorespiratory fitness of elderly healthy men who have undergone exercise training because this test lacks sufficient sensitivity. Particularly in OA, French et al. [20] studied the responsiveness of three physical performance measures of function following physiotherapy for knee OA and found that the 6-MWT was more responsive for the assessment of physical performance than the timed-up-and-go test and the timed-stand test.

Following 12 weeks of treatment procedures performed by the KIN and US groups, the distance covered in the 6-MWT increased by 19.8% and 14.1%, respectively. These improvements in functional exercise capacity indicate improvements in muscle strength and aerobic metabolism assuming that patients with knee OA are often physically deconditioned, interventions, as performed by current study, potentiate those muscle adaptations. Wang et al. [45] investigated the effects of aquatic exercises and land-based exercises for patients with knee OA and found that the 6-MWT performance increased by 19 ± 7% and 12 ± 5%, respectively. These changes were similar to the results found in previous studies [45,48]. Although these articles have studied different treatment modes, the results presented here suggest an improvement in functional exercise capacity and, consequently, of the quality of life and ability to perform activities of daily living. In fact, this assertion is supported by the positive results on the WOMAC and VAS scores. This improvement in ability to perform physical effort is very important because physical exercise is considered a valuable tool to reduce the risk of cardiovascular and endocrine diseases and to improve bone and muscle conditioning. These medical conditions may affect patients with OA due to the high level of inactivity and body disuse found in these patients. Indeed, this high level of inactivity can be demonstrated by the reduced aerobic capacity in patients with severe hip and knee OA compared to controls [49,50].

### Study limitations

We assessed study outcomes only on pre- and post-tests, so we were not able to determine the outcomes of these interventions across time. The evaluation of parameters related to exercise physiology, such as the maximal oxygen uptake, economy of motion and anaerobic threshold, could provide additional information on the level of aerobic fitness of the subjects before and after the treatment period.

## Conclusions

Many previous studies have compared one treatment protocol group with one control group and have concluded that the treatment made a difference, but there is no indication of how one program compares with other treatment protocols. Our study compared three popular non-pharmacological treatments. The main finding of this study was that the 6-MWT is a tool that can be used to evaluate improvements in the functional exercise capacity of patients submitted to a clinical intervention. Furthermore, the study results showed that KIN, TENS and US are effective for reducing pain and improving the WOMAC score and that KIN and US are effective for increasing the 6-MWT performance. Together, these results can be informative for both clinicians and patients with OA in selecting appropriate types of treatment based on their preferences and convenience.

The results of this study provide further evidence that patients with knee OA can achieve significant benefits from using KIN, TENS or US therapeutic procedures. The knowledge of function and disability for patients with knee OA, obtained through use of the 6-MWT, may help clinicians and physical therapists evaluate and develop rehabilitation programs to improve functional efficiency and capability for this population.

## Endnotes

The authors Claudio Andre Barbosa de Lira and Ibsen Bellini Coimbra contributed equally to this work and may be cited in interchangeable order.

## Abbreviations

6-MWT: 6-min walk test; KIN: Kinesiotherapy; OA: Osteoarthritis; ROM: Range of motion; TENS: Transcutaneous electrical nerve stimulation; US: Ultrasound; VAS: Visual analogical scale; WOMAC: Western Ontario and McMaster Universities.

## Competing interests

The authors declare that they have no competing interests.

## Authors’ contributions

NCM: study concept and design; data acquisition, analysis, and interpretation; manuscript preparation and critical revision of the manuscript. RLV and MdSA: data analysis, interpretation, manuscript preparation, and critical revision of the manuscript. EdPM: data acquisition and critical revision of the manuscript. CABdL: study and conception, manuscript preparation, and critical revision of the manuscript. IBC: study and conception, manuscript preparation, and critical revision of the manuscript. All authors read and approved the final manuscript.

## Pre-publication history

The pre-publication history for this paper can be accessed here:

http://www.biomedcentral.com/1471-2474/13/182/prepub
